# Drug resistance biomarkers in ovarian cancer: a bibliometric study from 2017 to 2022

**DOI:** 10.3389/fonc.2024.1450675

**Published:** 2024-11-11

**Authors:** Sindy Cabarca, Carmen Ili, Carlos Vanegas, Laura Gil, Melba Vertel-Morrinson, Priscilla Brebi

**Affiliations:** ^1^ Millennium Institute on Immunology and Immunotherapy. Laboratory of Integrative Biology (LIBi), Centro de Excelencia en Medicina Traslacional (CEMT), Scientific and Technological Bioresource Nucleus (BIOREN), Universidad de La Frontera, Temuco, Chile; ^2^ Grupo de Investigación Estadística y Modelamiento Matemático Aplicado (GEMMA), Departamento de Matemáticas, Facultad de Educación y Ciencias, Universidad de Sucre, Sincelejo, Colombia; ^3^ Semillero de Investigación (SIMICRO), Departamento de Biología, Facultad de Ciencias Naturales, exactas y de la educación, Universidad del Cauca, Popayán, Colombia; ^4^ Doctorado en Ciencia y Tecnología de Alimentos – Universidad de Córdoba, Montería, Colombia

**Keywords:** ovarian cancer, drug resistance, chemoresistance, biomarkers, descriptor, bibliometric analysis, biblioshiny, bibliometrix

## Abstract

**Background:**

Late diagnosis and patient relapse, mainly due to chemoresistance, are the key reasons for the high mortality rate of ovarian cancer patients. Hence, the search for biomarkers of high predictive value within the phenomenon of chemoresistance is vital. This study performs a bibliometric analysis of the scientific literature concerning biomarkers of drug resistance in ovarian cancer, considering the period from 2017 to 2022.

**Methods:**

The terms “drug resistance biomarker” and “ovarian cancer” were linked by the Boolean operator “AND”. The search was done in PubMed, selecting documents published over the last 5 years (2017-2022), which were analyzed with the open-source tool Bibliometrix developed in the R package. The language of the publications was restricted to English. Several types of papers such as case reports, clinical trials, comparative studies, and original articles were considered.

**Results:**

A total of 335 scientific articles were analyzed. The United States and China were the leading contributors and established the largest number of scientific collaborations. The Huazhong University of Science and Technology and the University of Texas MD Anderson Cancer Center were the most influential institutions. The Journal of Ovarian Research, International Journal of Molecular Science, and Scientific Reports are among the most relevant journals. The study identified high-profile, relevant thematic niches and important descriptors that indicate topics of interest, including studies on women, cell lines, solid tumors, and gene expression regulation. As well as studies involving middle-aged and adult participants, and those focusing on prognosis evaluation. Descriptors such as “drug resistance,” “neoplasm,” “genetics,” “biomarker,” “gene expression profile,” and “drug therapy” would indicate new research trends. In addition, we propose that BCL-2, CHRF, SNAIL, miR-363, iASPP, ALDH1, Fzd7, and EZH2 are potential biomarkers of drug resistance.

**Conclusions:**

This paper contributes to the global analysis of the scientific investigation related to drug resistance biomarkers in ovarian cancer to facilitate further studies and collaborative networks, which may lead to future improvements in therapy for this lethal disease.

## 1 Introduction

Ovarian cancer (OC) is the second most lethal malignancy of the female reproductive system. It ranks eighth in deaths worldwide and accounts for over 207,000 annually deaths (1, 2). Due to its imperceptible symptoms, early diagnose is difficult (3). Therefore, most of the time this disease is diagnosed at advanced stage, limiting treatment options thus highlights the importance of considering it a global public health problem (4). Besides, patients respond differently to chemotherapy depending on the cellular architecture, molecular profile, and genetic modifications of the type of OC they suffer (5).

The recommended treatment for this neoplasm includes a surgery and chemotherapy, mainly based on platinum drugs and paclitaxel (6, 7). However, patient relapse due to tumor resistance to platinum drugs is very common (approximately 75% of patients relapse during the first two years of treatment and develop chemotherapy resistance) (8) which is considered as the central problem to a better prognosis and also the main cause of low survival rates at advanced stages (20 – 30%) (8, 9).

Although some progress has been made in the genetic and epigenetic characterization of resistant tumors, biological and/or molecular parameters (biomarkers) that can be used in clinical practice to predict the response to platinum-based therapy (the primary therapeutic option currently available) have yet to be established (10–12). Consequently, identifying biomarkers with high predictive value for detecting drug resistance is crucial, since they aid in early diagnosis and also help to monitor treatment response, leading to improvements in available therapeutic alternatives and patient survival (13, 14).

Bibliometric analysis is a powerful and informative tool in basic research that provides a statistical framework for a systematic, transparent, and reproducible review of scientific information based on statistical measurements. Unlike other techniques, this method gives a more reliable and objective analysis (15). This study shows the results of a bibliometric analysis of the scientific literature published in the last five years related to drug resistance biomarkers in OC. The purpose was to identify current and relevant trends and patterns in scientific research resulting in high potential for future exploration and application; authors, studies, countries, and institutions with a high degree of contribution, among other useful data, when proposing future research, networking, and planning strategies to improve the treatment and knowledge of this lethal disease.

## 2 Materials and methods

### 2.1 Study design

The bibliometric method was used to analyze documents indexed in the PubMed database using qualitative and quantitative information from 2017 to August 2022, considering documents in “full text” and “free full” modes. For this reason, descriptors from the DeCS website (https://decs.bvsalud.org/E/homepagee.htm) were selected. We selected this period to analyze the most recent literature on biomarkers of drug resistance in OC. We are interested in the status, perspectives, and trends in this fascinating field of knowledge. In this study, the workflow recommended by Arias et al. was followed, in which data collection, analysis, and visualization were fundamental stages (15).

### 2.2 Search strategy

Scientific articles were searched using the terms “Drug resistance biomarker” and “Ovarian cancer,” linked by the Boolean operator “AND” in PubMed database, applying the filters “full text” and “free full text” and publications in the last 5 years. With this information, a database was created, and a bibliometric analysis was performed. Only scientific articles published in English were included, totaling 335 articles. The bibliometric indicators were title, year of publication, authors, subject areas, study type, affiliated country, institution, journal, collaboration network, and keywords. Documents such as early meeting abstracts, access, book chapters, retracted publication, proceeding paper, editorial material, letters, correction, and retractions, were excluded from the analysis.

### 2.3 Data analysis

The documents were exported from PubMed and saved as text documents (. txt format). We used R and R Studio (applying the Bibliometrix library) and Biblioshiny software packages for bibliometric analysis. This scientific mapping analysis tool provides several options for importing bibliographic data from scientific databases and performing comprehensive bibliometric analyses of any topic (16). All the statistical analyses were done. The data analysis had three substages. The first was descriptive for bibliographic data on articles, citations per article, authors, author appearances, authors of articles with one author - several authors, articles per author, coauthor collaboration, top 10 most productive authors, top 10 most cited articles, most productive countries–according to the affiliation of the first author–, most frequent journals, most frequent keywords, and citation analysis. The second stage involved the creation of networks to analyze bibliographic coupling (network), co-citation, collaboration, and co-occurrence. In the third stage, normalization was performed for which bibliometric taxonomy techniques were used for bibliographic coupling (author, document, and journal), co citation (author, reference, and journal), and co-occurrence data (author, country of affiliation, and institution of affiliation). Association (proximity index) was used as a measure of similarity (17). Following the PICO (Patient/population, Intervention, Comparison and Outcomes)model, our search focused on studies considering patients, animals, and cellular models or papers evaluating drug resistance in OC. The methods we were interested in were gene expression studies (RNA seq, microarray, qRT-PCR), gene silencing, protein inhibitions, and evaluation of the abundance/presence of proteins/mutations/molecules, highly associated with response to chemotherapy. Our controls were chemotherapy-sensitive patients/disease models. In this way, we were interested in studies that reported association, relevant correlation, and significant differences between molecules/chemoresistance and response to treatment/drug.

**Figure 1:**
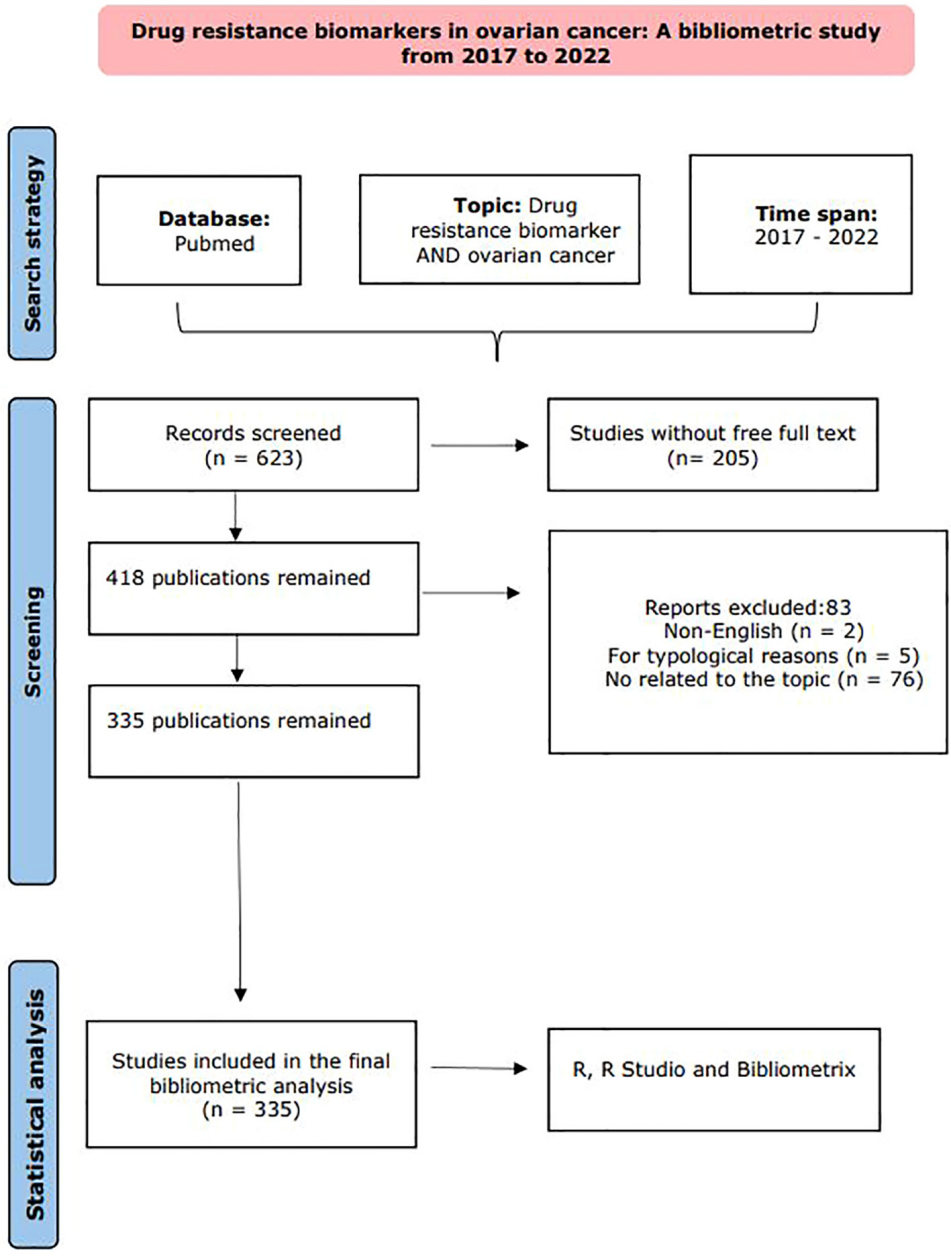
Flowchart of the exclusion and incorporation of literature. The overall workflow of the study includes three main stages: an initial Pubmed search for papers produced between 2017 and 2022 related to resistance markers in CO, followed by a screening stage, and finally, bibliometric analysis in R, R Studio, and Bibliometrics. Adapted from Page MJ, et al. BMJ 2021;372:n71. doi: 10.1136/bmj.n71.

Our procedural flow, from the initial data collection to the final article selection, is illustrated in the flowchart presented in **Figure 1**.

## 3 Results

### 3.1 Database analysis

The descriptors “Drug resistance biomarker” and “Ovarian cancer” used in this study come from a database of 335 documents published between 2017 and 2022, corresponding to research articles of various typologies (the main ones being case reports, clinical trials, comparative studies and original articles), which were used in their entirety in the analysis. The annual production evaluation started in 2017, with fewer than 20 articles. These results show a medium scientific output around resistance biomarkers, considering both the high relevance of OC and chemoresistance and the number of citations in the biomedical area of PubMed. During 2018 the number of papers tripled. From 2019 to 2021, the number of published works reached its maximum with an annual average of 80 articles. However, by 2022, this production suffered an abrupt drop with numbers like those observed for 2017.

### 3.2 Countries, institutions, and main collaboration networks

**Figure 2:**
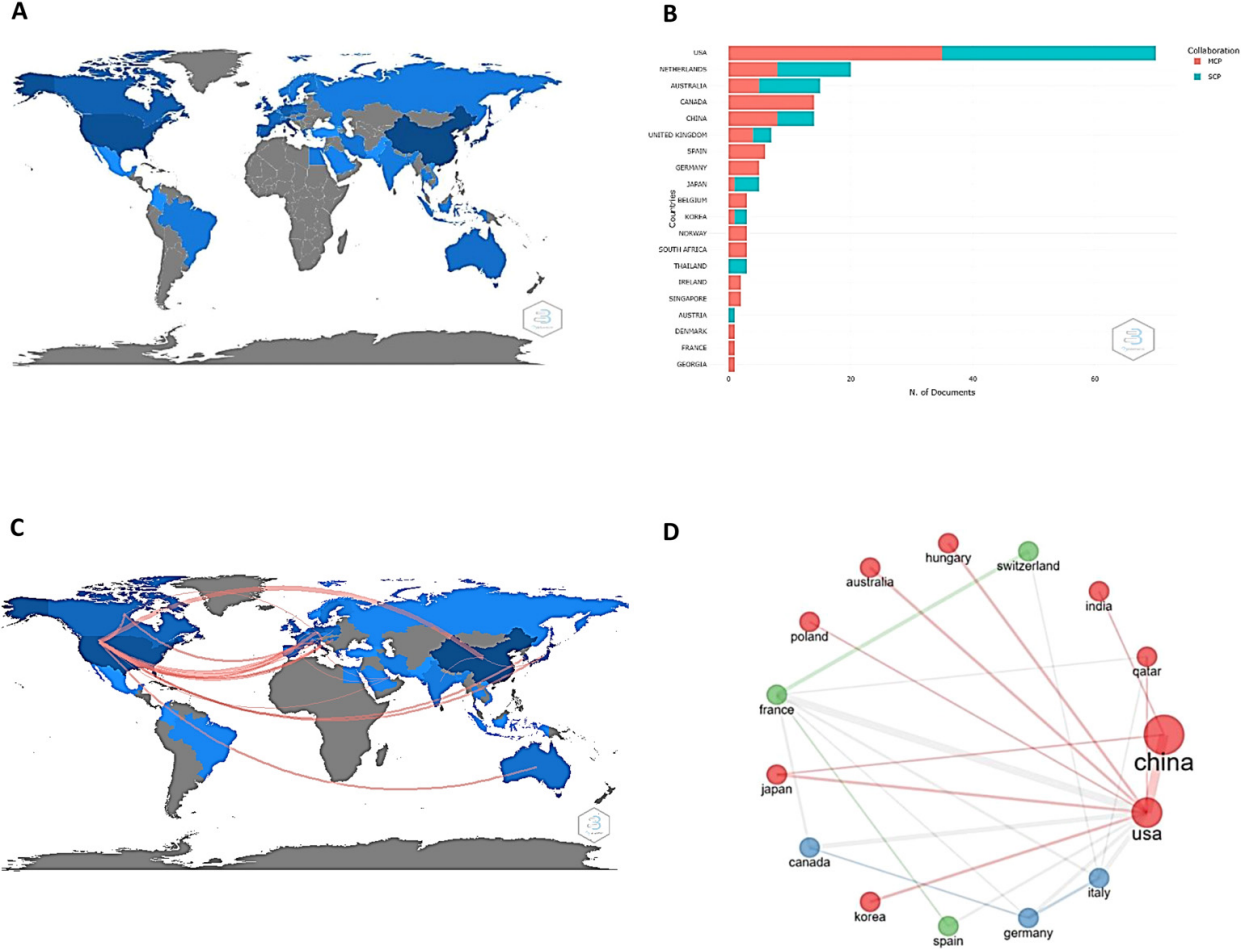
Scientific production and collaboration networks to research drug resistance biomarkers in ovarian cancer. **(A)** Worldwide scientific publications related to biomarkers of drug resistance in ovarian cancer. The most productive countries are shown in blue. **(B)** Internal (blue) and external production (red) of the 20 most productive countries in terms of published papers. *MCP*: *Multiple country publications, SCP: Single country publications.* **(C)** Main collaborations established between nations to work on research related to the subject of the present work. Remarkably, the workflow comes mainly from the United States. **(D)** Nations involved in research networks, reflecting the great network collaboration of China and the United States.

**Figure 2** illustrates the most productive and active countries in the field of study (2A), both internally (blue) and externally (red) (2B), based on the corresponding author’s affiliated country. The United States, Netherlands, Australia, Canada, and China are worth noting. Canada and China have similar outputs (approximately 20 works each), but only Canada presents its entire contribution externally.

**Figure 3:**
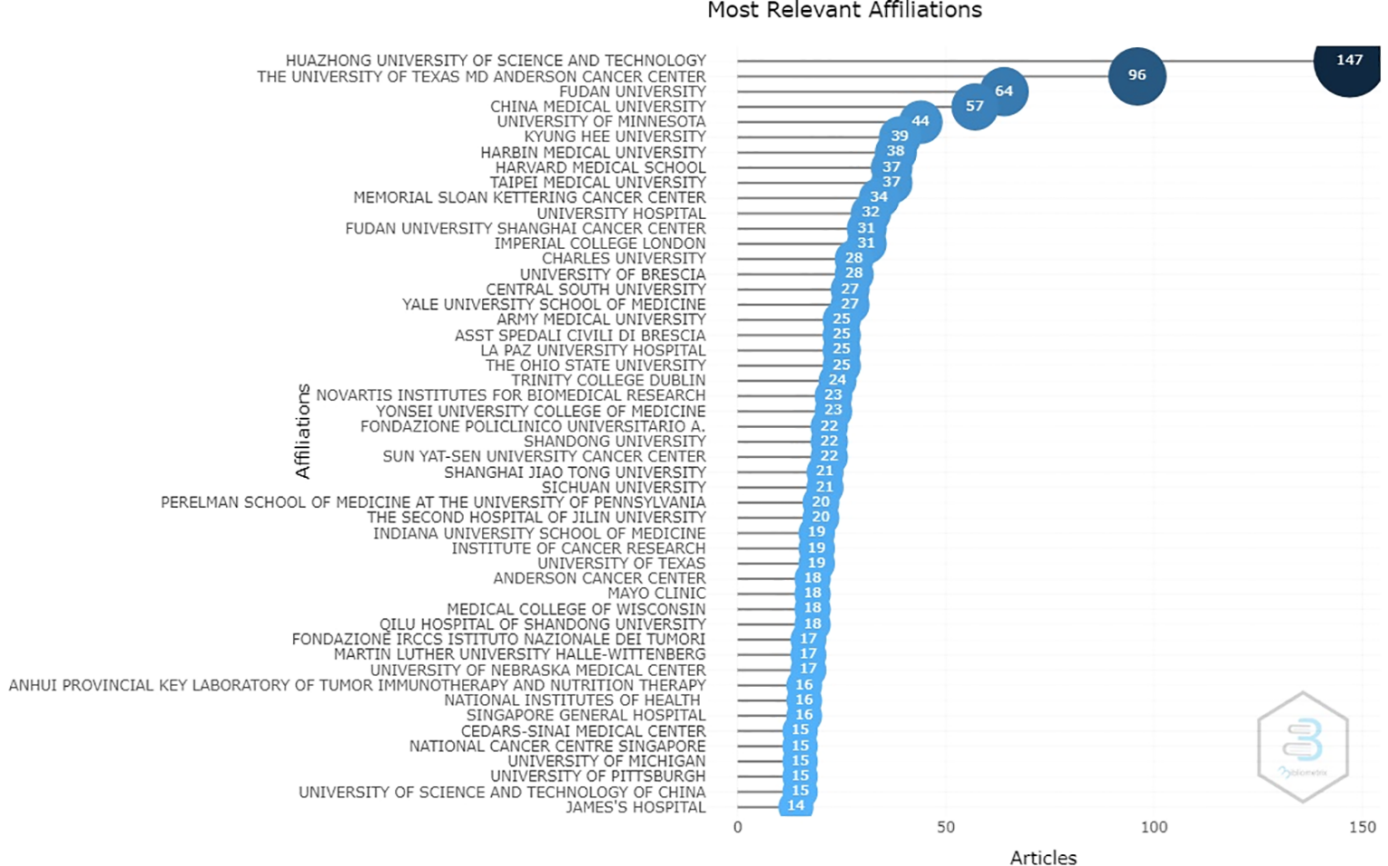
The scientific institutions with the greatest contributions to research related to biomarkers of chemoresistance in ovarian cancer (the number of articles in which each institution appears is shown next to it).

From an institutional perspective, the results show that a total of 50 institutions are mainly responsible for the scientific production of drug resistance biomarkers. The Huazhong University of Science and Technology (147), the University of Texas MD Anderson Cancer Center (96), Fudan University (64), China Medical University (57), and the University of Minnesota (44) are the most significant contributors considering the number of papers in which they appear (**Figure 3**). The results of this research show that four out of ten institutions are from United States and four are from China. In addition, China and the United States, establish many collaborations between them, and also collaborate with countries such as India and Japan (for China) and Korea, Japan, Poland, Australia, and Hungary (for the United States). This can be seen by the size of the nodes: the larger the nodes are the more productive the country. Collaborative work among these institutions is evidenced by the lines that connect them: the thicker the lines are, the greater the number of joint works.

### 3.3 Top contributing authors, articles, and journals

**Figure 4:**
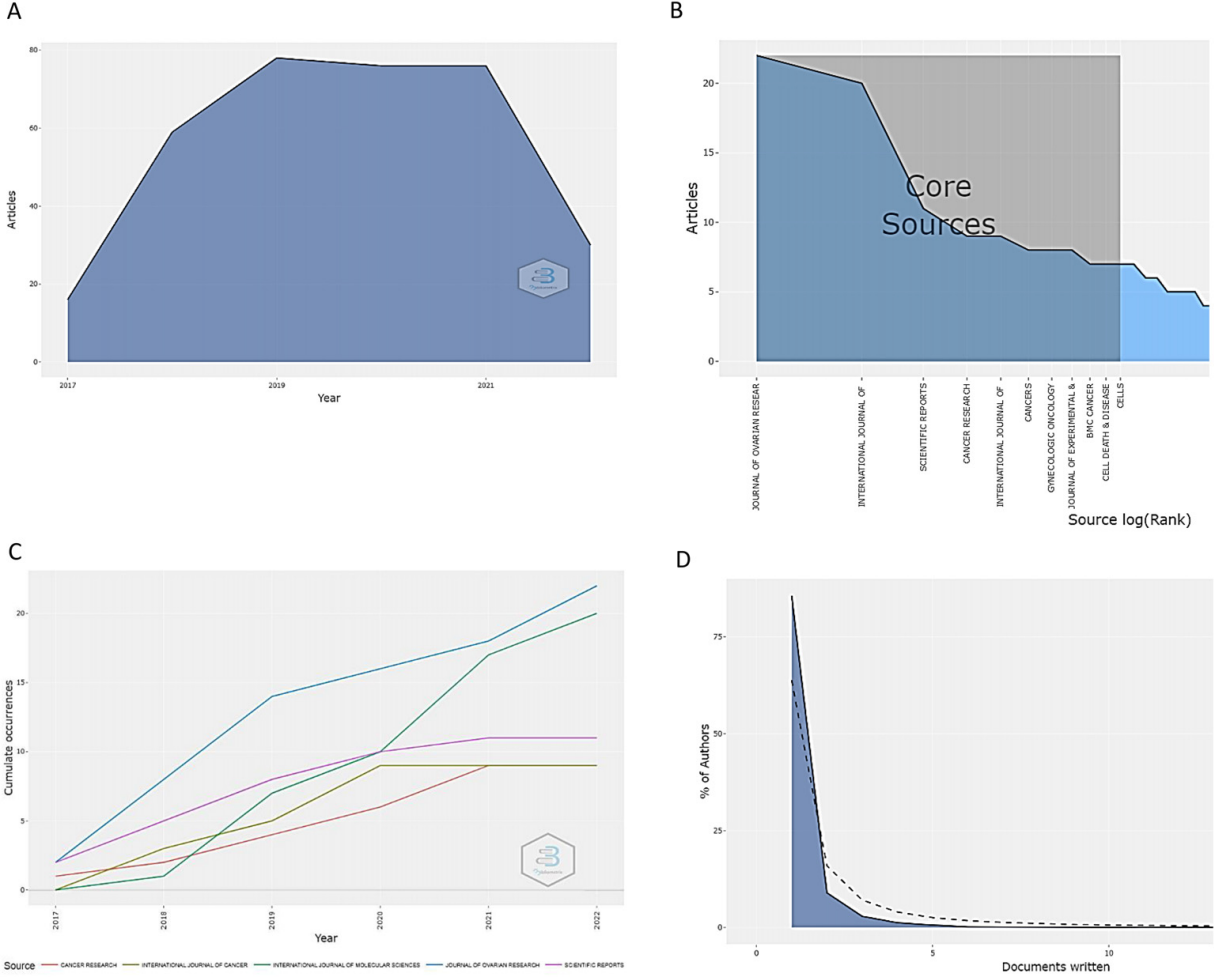
Scientific production from the last five years in research on drug resistance biomarkers in ovarian cancer. **(A)** Scientific articles published annually (2017–2022). Peak production occurred from 2019 to 2021 and was close to 80 articles. **(B)** Main sources of bibliographic information related to biomarkers of drug resistance in ovarian cancer. The Journal of Ovarian Research, International Journal of Molecular Sciences, and Scientific Reports lead in this sense. **(C)** Annual scientific production of the main journals in the field over the last five years. In general, there is a noticeable upward trend. **(D)**. Frequency distribution of scientific publications by author. More than 75% of the authors published a mean of one scientific article.

The analysis of the global scientific output related to drug resistance biomarkers in OC over the past five years revealed that the maximum production was reached between 2019 and 2021, with an overall output close to 80 papers per year (**Figure 4A**). After this period, there was an abrupt drop in production, perhaps because of COVID-19 pandemic. In general, the number of published papers per journal was between 1 and 22 for the most productive journals (**Figure 4B**). All these papers were published in 137 scientific journals. The journals with the greatest growth are the Journal of Ovarian Research (22), International Journal of Molecular Sciences (20), Scientific Reports (11), Cancer Research (10), and International Journal of Cancer (8). These journals are mostly area-specific or are closely related to the topic of our analysis. Part D of **Figure 4** displays another crucial piece of data, the proportion of authors for each specific number of papers. In that sense, 75% of the authors wrote one related article during the study period, which shows how challenging the production of articles in this field.

**Figure 5:**
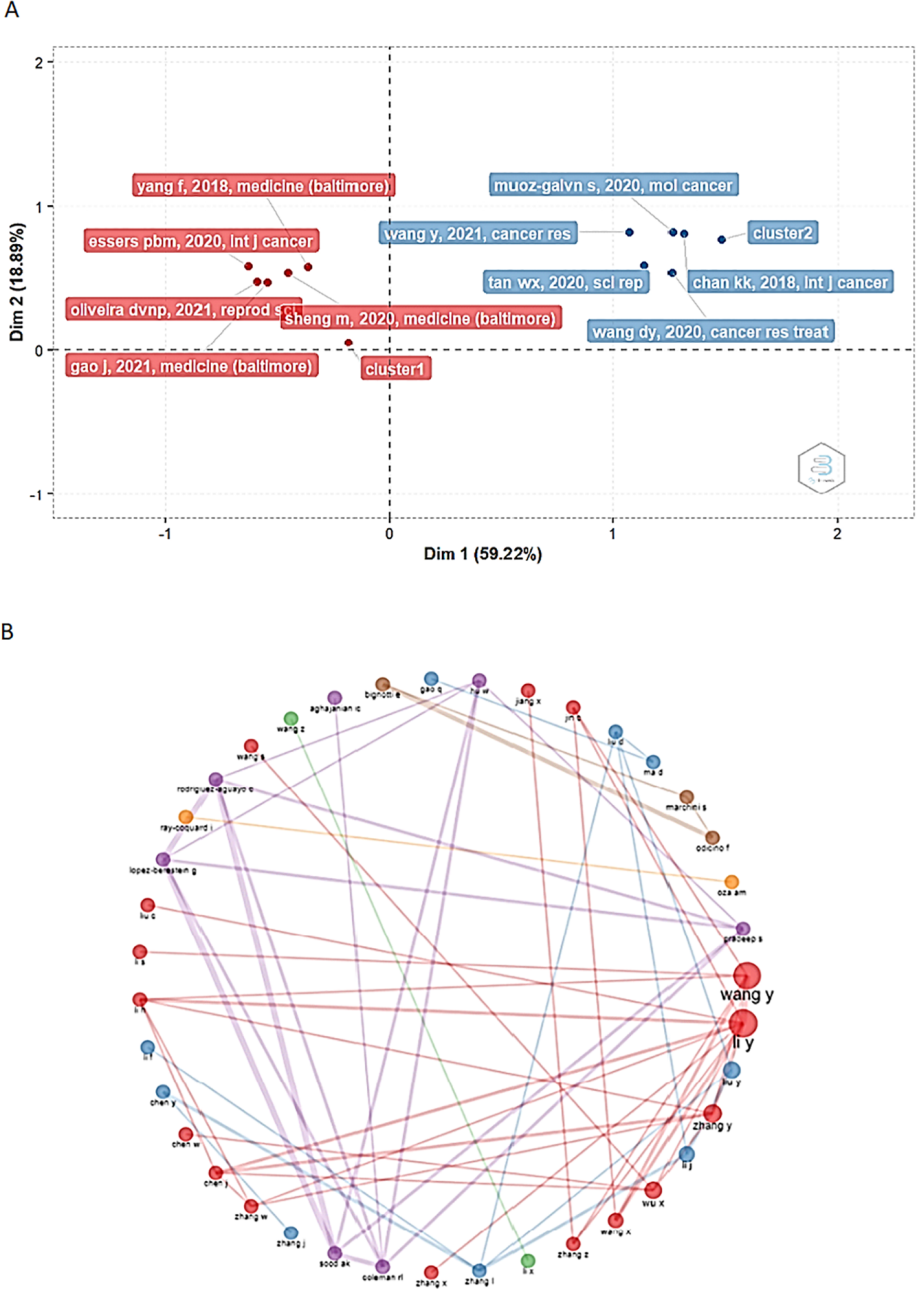
Factorial map and collaborative networks of the authors with the greatest contribution to this bibliometric analysis. **(A)** The analysis carried out to determine these results yielded an explanation percentage of 78.11%, showing two major clusters of authors (and their works) with the greatest contribution to the analysis. Dim1: Dimension 1 Dim2: Dimension 2. **(B)** Collaboration networks established among the authors of the scientific articles published around the interest of this work.

The review of the papers/authors with the highest number of contributions revealed two clusters, suggesting the existence of more connected research networks. Nine out of ten of the most influential documents, correspond to cluster 2 of the factor map (shown in blue in **Figure 5**). The papers with the strongest impact in the bibliometric analysis are presented in **Table 1** (details about these studies can be found in the table) and were written by Chan K, Muñoz-Galván S., Wang Y., Wang DY, and Tan WX (18–22). On the other hand, the five papers with the greatest contributions were published in the International Journal of Cancer, Molecular Cancer, Cancer Research, Cancer Research and Treatment, and Scientific Reports, which are among the ten most influential journals (**Figures 4B, C**).

**Table 1:** The ten papers with the greatest contribution to this bibliometric analysis.

Pos^a^	Title	YP^b^	Journal	Author	CR^c^
1	Impact of iASPP on chemoresistance through PLK1 and autophagy in ovarian clear cell carcinoma	2018	*International Journal of Cancer*	Chan, K. et al.	22
2	Downregulation of MYPT1 increases tumor resistance in ovarian cancer by targeting the Hippo pathway and increasing the stemness	2020	*Molecular Cancer*	Muñoz-Galván, S. et al.	41
3	Frizzled-7 identifies platinum-tolerant ovarian cancer cells susceptible to ferroptosis	2021	*Cancer Research*	Wang, Y. et al.	63
4	Long Noncoding RNA CCAT1 Sponges miR-454 to Promote Chemoresistance of Ovarian Cancer Cells to Cisplatin by Regulation of Surviving	2020	*Cancer Research Treatment*	Wan, D-Y. et al.	24
5	Novel role of lncRNA CHRF in cisplatin resistance of ovarian cancer is mediated by miR-10b induced EMT and STAT3 signaling	2020	*Scientific Reports*	Tan, W. et al.	21
6	EZH2 activates CHK1 signaling to promote ovarian cancer chemoresistance by maintaining the properties of cancer stem cells	2021	*Theranostics*	Wen, Y. et al.	15
7	Inhibition of miR-328-3p Impairs Cancer Stem Cell Function and Prevents Metastasis in Ovarian Cancer	2019	*Cancer Research*	Srivastava, AK. et al.	41
8	NRG1/ERBB3 Pathway Activation Induces Acquired Resistance to XPO1 Inhibitors	2020	*Molecular Cancer Therapeutics*	Miyake, TM. et al.	3
9	Protein kinase RNA-activated controls mitotic progression and determines paclitaxel chemosensitivity through B-cell lymphoma 2 in ovarian cancer	2021	*Oncogene*	Yin,L. et al.	0
10	Ovarian cancer-derived copy number alterations signatures are prognostic in chemoradiotherapy-treated head and neck squamous cell carcinoma	2020	*International Journal of Cancer*	Essers, PB. et al.	4

^a^Position; ^b^Year of publication; ^c^Cross referencing.

### 3.4 Thematic map of the scientific literature

**Figure 6:**
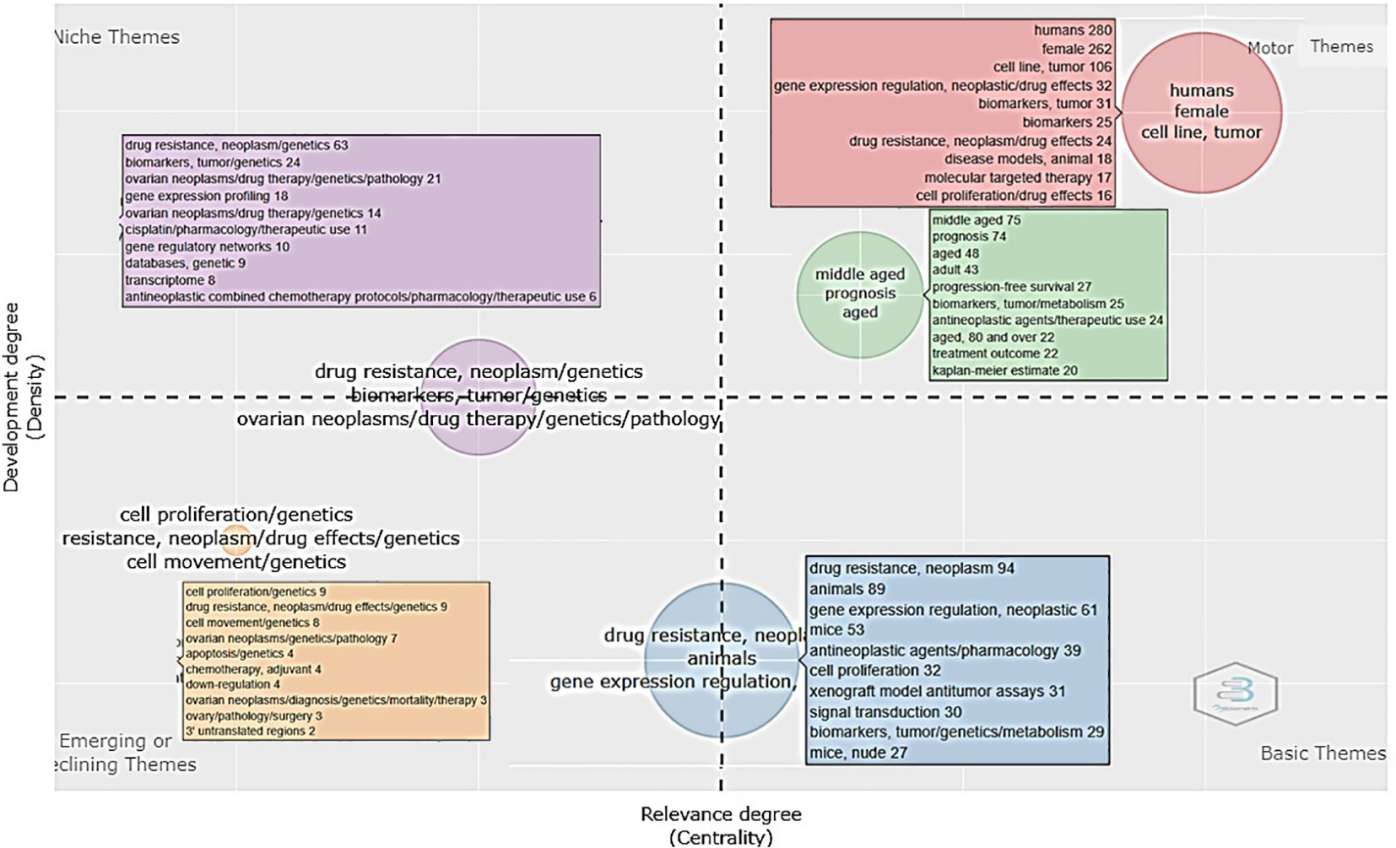
Thematic map of the scientific literature related to biomarkers of chemoresistance in ovarian cancer. Each thematic niche is presented in a different color. The motor themes are shown in red and green, the niches or isolated niches are shown in lilac, the emerging themes are shown in beige squares, and the basic niches are shown in blue. The numbers in parentheses indicate the frequency with which these descriptors appeared in the 335 papers analyzed (keyword plus section).

The thematic or niche map of scientific literature related to drug resistance biomarkers generates clusters resulting from co-occurrence, which are represented by two measures: centrality, which refers to the degree of interaction of one cluster with other clusters, and density, which refers to the internal cohesion of a cluster (17). The distribution of thematic niches in the four quadrants is shown in **Figure 6**. With this analysis it is possible to identify four focused themes: 1) motor (high centrality and density), meaning the themes are well developed and important for the structuring of the research field of interest, suggesting that the themes are fulfilled regularly and over a long period by a well-defined group of researchers. In this case, they are represented by two subclusters. The most frequent descriptors for the first subcluster are “human female,” “cell line,” “tumor,” and “gene expression regulation” and for the second subcluster are “middle-aged,” “prognosis,” “aged,” and “adult”. 2) Niche themes: The second quadrant corresponds to developed and isolated themes (high density and low centrality), implying that the importance within the field of resistance biomarkers is limited, as they do not share important external links with other themes. This may signal the emergence of a research question within a network that could be a motor theme in the future or a point of transfer between networks that are connected. In this case, the descriptors “drug resistance,” “neoplasm,” “genetics,” “biomarker,” “gene expression profile,” and “drug therapy” are included in this group. 3) Third quadrant: emerging or disappearing themes (low centrality and density), suggesting underdevelopment and marginality. The descriptors “cell proliferation/genetics,” “drug resistance,” “neoplasm,” “drug effects,” “cell movement,” “apoptosis,” “ovarian neoplasm,” and others were identified in this cluster as the most frequently co-occurring. 4) Finally, the fourth quadrant (high centrality and density) represents basic themes of general importance and significance to different research areas/domains in the field. In our map they would be represented by descriptors such as “drug resistance,” “neoplasm,” “animals,” “gene expression regulation,” “neoplastic,” “mice,” and “biomarkers,” among others.

**Figure 7:**
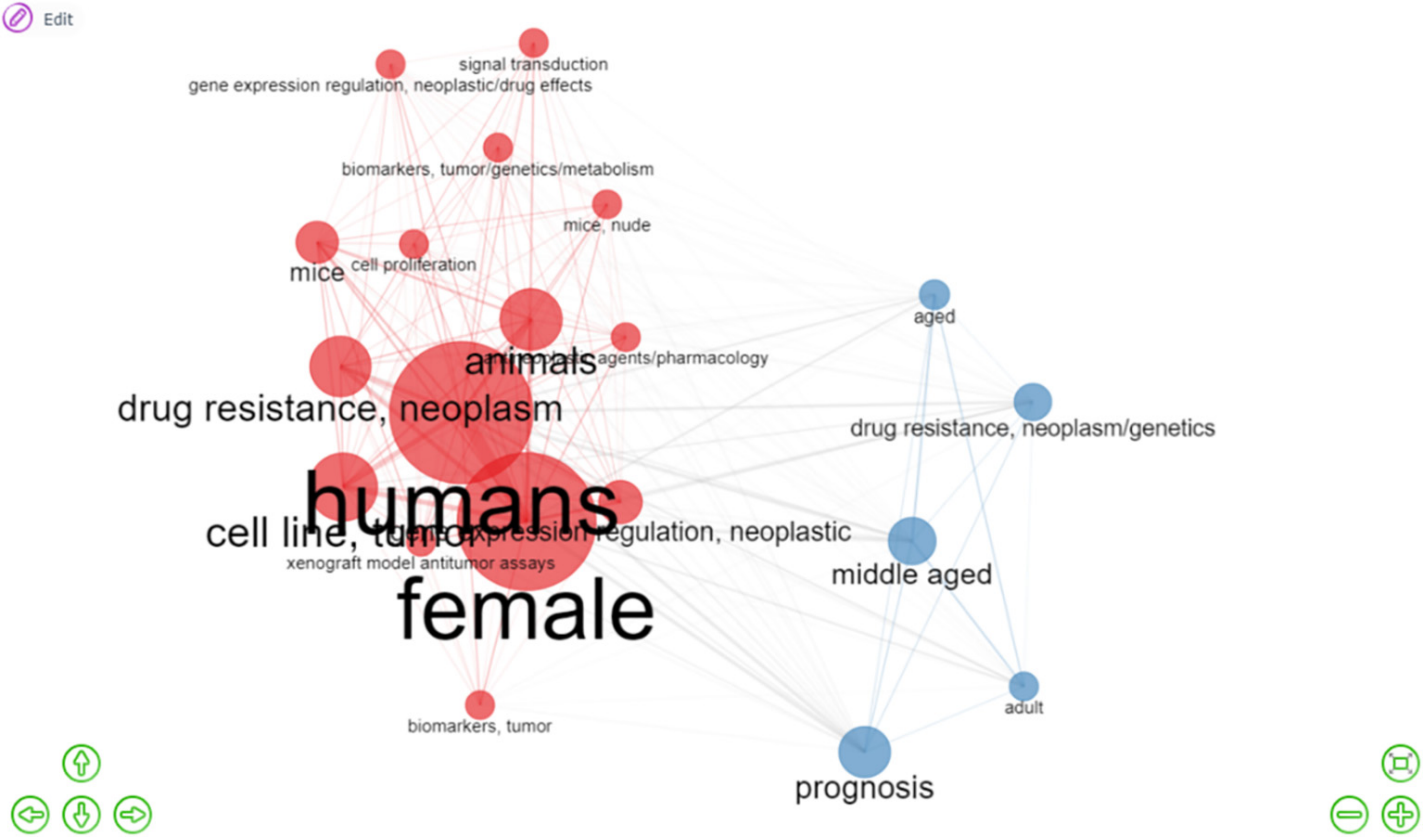
Interaction network (co-occurrence) among the most relevant descriptors. The results show two large networks (red and blue) connected to each other, obtained from the most frequent descriptors in this analysis. The descriptors in red and in larger spheres correspond to the most relevant words and are related to the terms “human,” “female,” and “drug resistance/neoplasm”. In contrast, the descriptors in blue refer to slightly less frequent descriptors associated with “prognosis” and “middle-aged,” suggesting emerging research areas.

The word network (**Figure 7**) also shows the conceptual structure of the association between concepts and descriptors based on their co-occurrence. In this case, the network has 20 nodes, divided into two groups, shown in red and blue, which are related to the most frequent themes/descriptors and their relationships between them (indicated by the extension of their edges: the shorter the edges are, the closer the nodes or research topics are). The strength of these relationships is determined by the thickness of the edges that connect them. Furthermore, larger nodes represent a greater frequency of occurrence or presence in the analysis, whereas smaller nodes reflected themes with a lower frequency of occurrence and suggested emerging trends within the research area. **Figure 7** shows two groups (one in red and the other in blue), which suggest two interconnected research areas. First, there are a large number of papers related to the words “humans,” “female,” “cell line,” “tumor,” “drug resistance,” “neoplasm,” and “animals,” following the basic and traditional line of research. On the other hand, there is a predominance of papers associated with the descriptors “prognosis,” “middle-aged,” “drug resistance” and “neoplasm/genetics,” which in turn suggest emerging research topics.

### 3.5 The most relevant words

**Figure 8:**
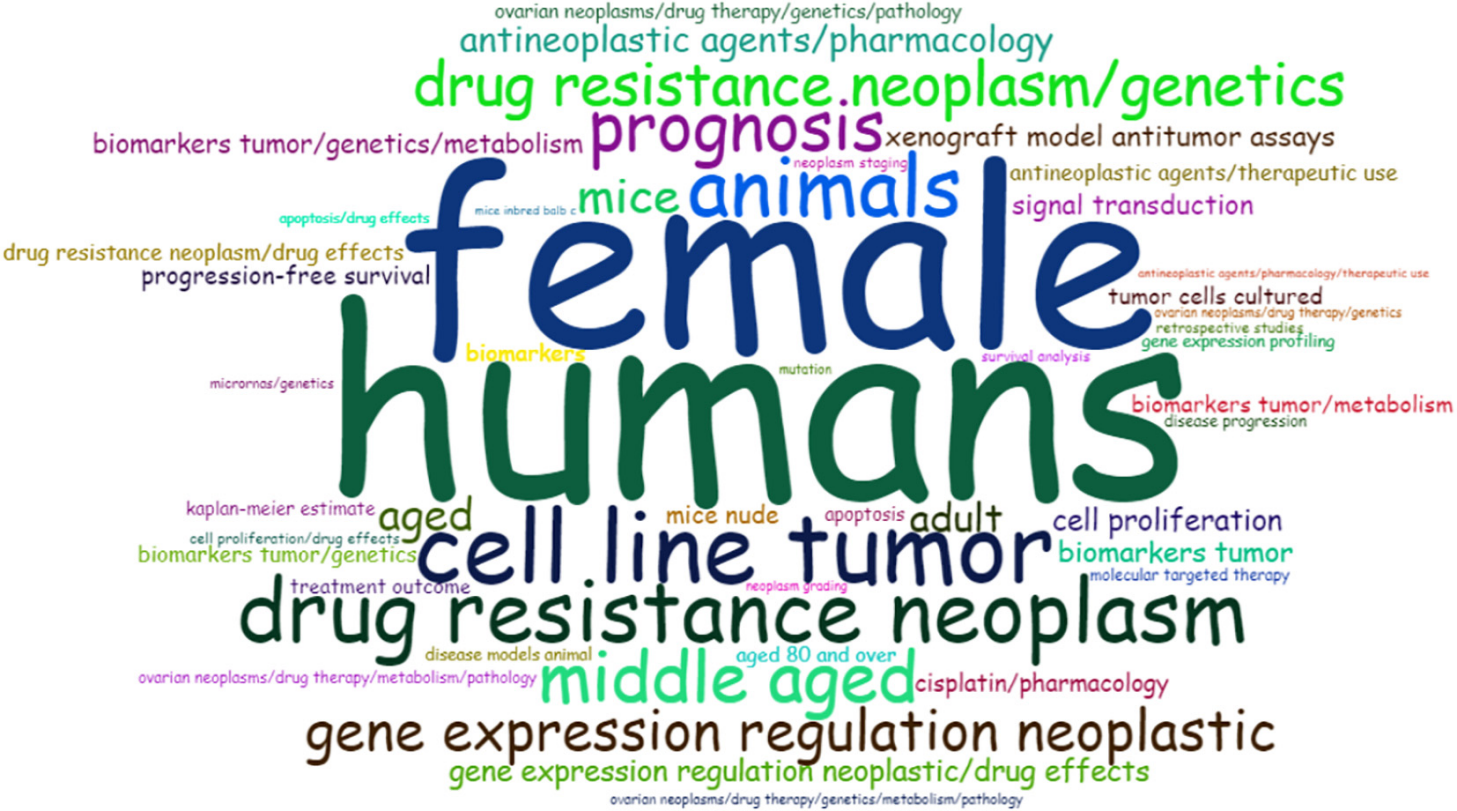
Occurrence of keywords using *Word Cloud* visualization. Most frequently occurring words in papers related to biomarkers of drug resistance in ovarian cancer. The size of the words indicates the greater or lesser frequency with which they appear.

The analysis of the most relevant keywords (keywords plus) revealed that the words “human” (198), “female” (130), “male” (114), “adult” (93), “young adult” (85), adolescent (83), “middle-aged” (62), and “child” (56) appeared much more frequently in the analysis (**Figure 8**). In the abstract, the most frequently used words were “cancer” (1230), “ovarian” (1076), “patients” (652), “expression” (536), “cells” (528), “resistance” (514), “cell” (396), “treatment” (331), and others. However, it is worth noting that some less frequent words, such as “biomarkers” (157), “gene” (152), “platinum” (147), “cisplatin” (144), and “inhibitors” (144), are beginning to emerge as new keywords, suggesting the novelty and timeliness of this topic and emerging lines of research within searching for chemoresistance biomarkers in OC.

### 3.6 Our proposal for biomolecules with potential use as biomarkers of drug resistance in OC

Based on a review of the 30 articles with the greatest contributions to the study, we propose a list of biomolecules with high potential as predictive biomarkers in the phenomenon of drug resistance in OC (**Table 2**). This was done considering the main results achieved by the study, the evaluated models, the detection and correlation with patient samples and the progress in the validation process. For this reason, we propose eight biomolecules for future exploration and validation as biomarkers of chemoresistance. Six correspond to proteins involved in multiple biological processes. The remaining two are a micro-RNA (miRNA) miR363 and a long noncoding RNA (LncRNA CHRF (Cardiac hypertrophy related factor). As shown in **Table 2**, the proteins for which we found the most promising results and advances are BCL-2 (B cell lymphoma 2 protein), SNAIL (Zinc finger protein SNAI1), iASPP (Inhibitor of apoptosis-stimulating protein of p53), ALDH1 (Aldehyde dehydrogenase 1A1), FZD7 (Frizzled-7), and EZH2 (Enhancer of zesta homolog 2). These proteins participate in various biological processes such as apoptosis, epithelial-mesenchymal transition, oncogene regulation, retinol metabolism, cell signaling and gene repression.

**Table 2:** Biomolecules with potential use as predictive drug resistance biomarkers in ovarian cancer proposed in this study.

Name	Response to	Function	Model	Promising results	Ref^d^
**BCL-2** (Protein)	Cisplatin and Paclitaxel	Regulation of apoptosis	Xenograft mice, cells line SKOV3, MCF7, TOV21G	-BCL-2 inhibition overcomes paclitaxel resistance *in vivo.* - More reduction in weight and caspase-3 positive cells cleaved from pc-SKOV3 xenograft tumors compared to *Bcl-2*-SKOV3 xenograft tumors, following cisplatin treatment.	(78, 79)
**CHRF** (LncRNA)	Cisplatin	Regulation of miR-10b	ES2, OVCAR, SKOV3 cells and drug-resistant patient tissues.	-Elevated CHRF levels in tissue samples and cisplatin-resistant cells. - CHRF down expression reverses cisplatin resistance.	(22)
**SNAIL** (Protein)	Cisplatin	Induction of the EMT (epithelial-mesenchymal transition)	Cells A2780, C13, OV2008, and tissue.	- Snail overexpression in cells and patients with cisplatin resistance.	(99)
**miR363** (Mi-RNA)	Cisplatin	Regulation of gene expression.	A2780, C13, OV2008, xenograft, and tissue.	-Low expression in chemoresistant patients and cisplatin-resistant cell. - Sensitization to cisplatin following restoration of miR363 expression.	(99)
**iASPP** (Protein)	Cisplatin	Regulator of key proteins such as p53 and p63.	OVTOKO and KK cell lines; xenograft tumors.	- iASPP expression correlates with chemoresistance and poor survival in patients. - Silencing of iASPP resensitizes xenograft cells and tumors to cisplatin.	(18)
**ALDH1** (Protein)	Platinum, Cisplatin and Paclitaxel	Aldehyde dehydrogenase	Organoids and clinical samples	-Increases in cisplatin resistance related to increases in ALDH levels. -ALDH inhibitors significantly reduces the viability of CSC spheroids resistant to cisplatin. -ALDH1 expression decreases after gradual use of cisplatin. -It was one of the overexpressed genes related to chemoresistance.	(20, 90)
**FZD7** (Protein)	Platinum and Cisplatin	Cell receptor for Wnt proteins. Regulator of pluripo- tency	Human ovarian high-grade serous cancer tissue, cell lines: SKOV3, OVCAR3, OVCAR5, COV362, PEO1, and PEO4. Xenografts	-*FZD7* deletion increased platinum sensitivity, decreased spheroid formation, and delayed tumor formation. - FZD7 expression activated the oncogene *Tp63* and metabolic pathways to protect against chemotherapy. - FZD7 expression correlates with residual OC^e^ after chemotherapy.	(20, 100)
**EZH2** (Protein)	Platinum	Induces transcriptional repression of target genes.	OC^e^ stem cells (SK-3rd), clinical samples, cell lines (SKOV3, A2780, IGROV1, OVCAR4).	-EZHZ contributes to maintenance of *stemness* and chemoresistance. -Patients with high EZH2 expression were more resistant to platinum treatment and had worse prognosis.	(77)

^d^References; ^e^Ovarian cancer.

These results are interesting because they were obtained by evaluating several disease models, including various OC cell lines, xenograft mice, organoids, and clinical samples, providing solid scientific support for the findings obtained. In addition, as can be seen in column 5 of **Table 2**, most of the results of these works are going to the over expression or high abundance of the molecule of interest in models and patients resistant to cisplatin, as well as the restoration of sensitivity to the drug after down-regulation of the gene(s) involved.

## 4 Discussion

This study presents a bibliometric analysis of biomarkers for drug resistance in OC. The results show medium scientific production in the area and suggest that it is a modern (emerging) topic of broad and interdisciplinary significance, led mainly by the United States and China and renowned institutions such as Huazhong University of Science and Technology and the University of Texas MD Anderson Cancer Center. The USA’s leadership is a reflection of the significant investment that this nation makes in cancer research (57.3% of the total investment) (23). On the other hand, China is the nation with the highest scientific collaboration activity in the research of drug resistance biomarkers in OC. Both have four of the ten most influential institutions in the field. These results demonstrate the two great strategies for success in the evolution of any area of scientific knowledge: investment and collaboration. Remarkably, no Latin American institutions or countries appeared in the entire analysis, which shows the region’s underdevelopment and relegation despite the high prevalence of the disease (24). By comparing our results with those obtained by Duan et al., who analyzed the scientific productivity of platinum-resistant OC (a larger area), we observed that from 2017 to 2022, the number of annual articles ranged between 200 and 250 (25). In our case, for 2017, the number of papers related to biomarkers of drug resistance was less than 20. However, for the period from 2018 to 2021, this number was approximately 80 papers per year, which represents 32 to 40% of the research in chemoresistance, denoting the importance and novelty that this line of research has within this phenomenon.

Another interesting result relies on the most influential authors because, as shown in the study, more than 75% of the authors contributed with a single paper and 20 of them accounted for 63% of the works included here and the scientific productivity evaluated. This finding suggests the highly challenging and restrictive nature of this area of research in terms of human capital. Regarding the journals, the Journal of Ovarian Research, International Journal of Molecular Science, Scientific Reports, Cancer Research and International Journal of Cancer are the most influential, followed by others of high quality and related scope such as Gynecologic Oncology, Journal of Experimental and Clinical Cancer Research, PLoS One and Cells. These are all high-impact journals (with a 5-year impact factor higher than 4) and of high scientific quality with a scope mostly related to cancer research, molecular biology, biochemistry, biomedicine, gynecology and obstetrics. All are Q1 in the fields directly related to their scope (oncology, biochemistry, gynecology obstetrics). These show the high relevance and impact of research associated to drug resistance biomarkers in OC, and how challenging it is to publish articles in this area.

By reviewing the highest contribution articles is possible to notice that these papers evaluate the role of a particular biomolecule(s) in the chemoresistance in various models and samples of OC (cell lines, patient samples, organoids, animal models, xenogeneic tumors, etc.) Most of these studies present a high number of cross-citations and have promising results in the search for biomarkers of resistance. As part of the analysis of the results obtained in these and 30 other articles, we extracted the list of biomolecules that present high potential as biomarkers of chemoresistance, which, as shown **Table 2**, involve mainly proteins that participate in many cellular processes, such as the regulation of apoptosis, the induction of epithelial-mesenchymal transition, the regulation of other proteins and cell signaling. The participation of molecules, like long non-coding RNAs and micro RNAs, is also demonstrated by the related scientific literature. The usefulness of each of these molecules is discussed below.

The examination of themes and trends in the literature, because of the evaluation of frequency and co-occurrence of keywords, allows us to have an overview of the general panorama of the field of the study concerning resistance biomarkers in OC. From this we can observe the main research trends that an area of interest is following and evaluate the future perspectives. In that sense, we present a thematic map of research related to resistance biomarkers in OC. We identified four main thematic clusters (**Figure 6**) in which the central topics are represented by descriptors that illustrate basic research directions, considering the biology and epidemiological characteristics of the disease. Therefore, keywords such as “humans,” “female,” “cell line,” “tumor,” “gene expression regulation,” etc., were often used. There is a second niche of motor topics that is more related to prognosis and includes descriptors like “middle-aged,” “prognosis,” “aged,” “adult,” “progression free survival,” and “biomarkers”.

The descriptors closely related to the topic of this investigation, i.e., words like “biomarker,” “drug resistance,” “platinum,” and “gene expression,” results to be less important in the motor themes quadrant on the thematic map and appear in greater abundance in quadrants two and four (**Figure 6**), which contain themes that have the potential to became future trends and of interdisciplinary significance to multiple fields of research. They also appear as smaller spheres in the interaction network and in the occurrence graph shown in **Figure 7**. This suggests that the search for chemoresistance biomarkers in OC is a relatively new trend of increasing relevance. Considering the above, it is important to note that, as revealed by various studies in the area and our results, the current and perhaps classic trend in this disease research continues to be studies conducted on women, cell lines, tumor samples, and adults due to the nature of the disease. Nevertheless, modern and prevailing research involves the search for biomarkers, the study of drug resistance, gene expression and transcriptomic. Thus our results are very similar, and we agree with those obtained by Duan et al., who observed that the keyword “biomarker” defines one of the most recent and significant aspects of research related to platinum-resistant OC. This work has similar results to those obtained by Duan et al. in terms of countries, institutions and most influential journals in the area, which evidence the reproducibility and objectivity of our conclusions (25).

The search for biomarkers in OC is performed following multiple approaches ranging from DNA sequencing, gene expression evaluation, and proteomic analysis to validation in patient cohorts. In this regard, the best and most extensively studied association between molecular markers and the response to chemotherapy is related to *BRCA* (Breast cancer susceptibility) genes. Additionally, the expression of several proteins (such as P-glycoprotein (P-gp), CA125 (Cancer antigen 125), HE4 (Human epididymis protein 4), CHK2 (Serine/threonine protein Kinase Chk2), NOTCH3 (Neurogenic locus notch homolog protein 3), AEG1 (Astrocyte elevated protein 1), XIAP (X-linked inhibitor of apoptosis), CD133 (CD133 antigen), VEFG (Vascular endothelial growth factor), PD-L1 (Programmed cell death protein ligand 1) and CA9 (Carbonic anhydrase 9), miRNAs(mi-R9, miR-21-3p, miR-181a, and miR-106a) and transcription factors (NF-KB (Nuclear factor kappa B)), mutations in certain genes (*BRCA1*, *BRCA2*, *TP53* (Tumor antigen p53), HRD (Homologous recombination deficiency) level and alterations in DNA methylation patterns (homeobox A9 (*HOXA9*) promoter) have been associated with resistance to chemotherapy in OC and have shown great potential in the evaluation of this phenomenon. None of them has been used in clinical practice for the follow-up, monitoring, or treatment management of patients with chemoresistant OC (only *BRCA* and CA125 are used, but its actual application is limited) (13, 26–29). Because of many of them has been made a considerable advance and some level of application as biomarkers of resistance has been achieved. In this article, we discuss the most relevant, and some of the most important findings for each of them.

P-gp is an ABC Transporter, which translocate drugs and phospholipids through the membrane. It is one of the central molecules mediating multidrug resistance in cancer (30). In OC, it participates in paclitaxel resistance, both of them, paclitaxel-resistant cell lines (A2780) and patient samples overexpress this protein (31). In addition, patients with higher expression of P-gp and MRP1 (Multidrug resistance protein 1) proteins had a higher frequency of relapse during the 24 months following the start of therapy compared to patients with lower expression, which has also been associated with poor survival (32). Sharma et al. reported sensitization of highly paclitaxel-resistant cells (NCI/ADR-RES) using the P-gp inhibitor, PGP-41, which decreased the paclitaxel IC50 from 6.64 μM to 0.12 μM in these cells (33). Zhang et al. reached similar results. Who also reversed paclitaxel resistance in several CO-resistant cell lines using a P-gp inhibitor (XR-9576, XR) (34).

HRD is the first defined phenotypically predictive biomarker for PARP inhibitor therapy in High-grade serous carcinoma (HGSC). HRD is caused by pathogenic germline/somatic variants and epigenetic modifications in either *BRCA1/2* or essential genes in the HRR (Homologous recombination repair) pathway (35, 36). In this regard, the most known mechanism of Poly-ADP-ribose polymerase inhibitors (PARPi) resistance is the reversion of HRD to homologous repair proficiency (HRp) driven by epigenetic and genetic changes (37). Approximately 50% of HGSC cases do not have HRD, which makes them more prone to PARPi primary resistance (38). Clinical trials for PARPi PRIMA (niraparib), PAOLA1 (olaparib), and VELIA (veliparib) have shown better benefits in patients with pathogenic *BRCA* mutations (*BRCAmut*) and patients with *BRCA* wild type but HRD deficient (*BRCAwt*/HRD) relative to those with proficient HR (HRp). The FDA has approved trials to assess HRD in platinum and PARPi drug therapy. In this context, the most widely used test is MyChoice CDx (Myriad Genetics, Salt Lake City, UT, USA), which calculates a Genomic Instability Score (GIS) based on the evaluation of loss of heterozygosity (LOH), telomeric allelic imbalance (TAI), and large-scale state transitions (LST). Furthermore, *BRCA1/2* variants are analyzed. Tumors with GIS < 42 are HRp, and tumors with GIS ≥ 42 and/or pathogenic *BRCA1/2* variants are HRD (39). Despite all this evidence, there is an urgent need for improved biomarkers to HRp evaluation and the subsequent stratification of HGSC management (40).

*BRCA* genes (1 and 2) are tumor suppressor genes that play substantial roles in maintaining genomic stability. Cells with germline mutations in these genes are inefficient in the DNA double-strand break reparation by homologous recombination (HR). Pathogenic variants of these genes correlate with increased response (up two-fold higher) to platinum-based chemotherapy and sensitivity to PARPi primarily (41, 42). Many studies address PARPi therapy according to *BRCA* mutations (43–45). For example, Niraparib is a selective PARP-1 and PARP-2 inhibitor with favorable pharmacokinetics and anti-tumor roles in patients with *BRCA1/2* mutations. In patients with *BRCAmut* or *BRCAwt* OC treated with rucaparib (a PARPi) as therapy after platinum-based therapy for patients of platinum-sensitive and recurrent OC, PFS (Progression-free survival) and OS (overall survival) was more extended than patients without *BRCAmut* OC (Guo et al., 2020).

Other trials have been conducted to identify predictive biomarkers to monitor response to PARPi and other drugs in OC beyond HRD and *BRCA* status. That is the case of the P53-binding protein, 53BP1, whose low levels correlated negatively with response to PARPi Veliparib ABT-888 and ABT-767 in OC with HRD and deleterious *BRCA* mutations. Similarly, methylation of the *HOXA9* promoter from circulating tumor DNA was successfully used to PARPi-based therapy in patients with platinum-resistant OC and germline *BRCA* mutations (46, 47). MAPK (Mitogen- activated protein kinases) pathway gene alterations can also be useful in combinatory regimens with MAPK inhibitors (48).

HE4 is mainly expressed in the respiratory and reproductive tracts but is over-expressed in OC. This protein can effectively predict resistance to platinum-based therapy in OC (49–51). In OC cell lines (SKOV3 and OVCAR), its overexpression decreases the cisplatin and paclitaxel sensitivity, as well as the expression of apoptosis-promoting genes PARP and EGR1 after treatment with these drugs (52). Wang et al. has similar results but in response to carboplatin (53). In patients, data support its use as a chemotherapy resistance biomarker and worse prognosis (54, 55). FDA approved it in OC monitoring. However, there is still a lack of standardized guidelines for its application in clinical practice (50).

CA125 or mucin 16 (MUC16) has been a reliable biomarker used in OC screening. FDA recommends its use in treatment response evaluation and residual disease monitoring or risk of recurrence after the first-line regimen. Several studies show that CA125 is an accurate progression predictor after first- and second-line chemotherapy (56, 57). Serum levels of this antigen allow monitoring outcomes in patients with HGSC (58). The early biomarker of tumor chemosensitivity, KELIM (The modeled CA-125 ELIMination rate constant K), has been validated for patients with stage III or IV HGSC treated with adjuvant carboplatin-paclitaxel regimen and is calculated with the CA125 longitudinal kinetics during the first 100 chemotherapy days (59). Monitoring its clearance and HE4 protein predicts resistance to platinum-based drug treatment with 50 and 94.5% sensitivity and specificity, respectively (60).

VEGF proteins family is the major component in maintaining the vasculature in physiological and pathological conditions. This family includes VEGF-A, VEGF-B, VEGF-C, VEGF-D, and PIGF (Placental growth factor) proteins (61). A recent study by Li et al. shows that treatment of SKOV3, A2780, and CAOV3 cell lines with cisplatin (DDP), adriamycin, and paclitaxel increases expression at least 2-fold of VEGF-A factor and correlates with chemotherapy resistance. Deletion of the gene coding for VEGF-A promotes sensitivity to chemotherapeutic drugs in cisplatin-resistant lines. Authors suggest results may reflect the cells’ adaptive response to drug stress and damage (62). The FDA approved the use of the anti-VEGF monoclonal antibody in patients with platinum-resistant OC, and other anti-VEGF agents also show efficacy when combined with chemotherapy in platinum-resistant patients. Pre-clinical evaluation of the anti-angiogenic and anti-metastatic agent PG545 increases serum VEGF levels in pre-clinical models and a small group of patients with advanced cancer, suggesting it may be a biomarker of anti-angiogenic response (63).

PD-L1 is the principal ligand of PD-1 (Programmed cell death protein 1) and is expressed mainly by tumor and immune cells. Its over-regulation stimulates its binding to PD-1 on the surface of T cells, thereby inhibiting the effector function of local T cells, allowing tumor cells to evade the immune system (64). A meta-analysis to evaluate the prognostic role of its expression in patients with HGSC found that expression of this protein by immune cells correlates positively with patient survival (HR = 0.73, 95% CI: 0.55-0.97, p = 0.031). The presence of PD-1+ TILs (tumor-infiltrating lymphocytes) improves OS and PFS (65). These results are similar to those reported by Buderath et al. and Weeb et al. (66, 67). Meanwhile, Świderska et al. report that serum and peritoneal fluid PD-L1 concentration is an unfavorable prognostic factor for OC (68). Clinical trial evaluation of PD-L1 as a biomarker of response to therapy has been associated with favorable results among patients with HRR mutations treated with olaparib + durvalumab, where patients with high PD-L1 expression (CPS ≥ 10) had better PFS compared to those without (69). Other clinical trials also show the benefit of adding an immune checkpoint inhibitor to PARPi therapy in patients with high PD-L1 levels (70, 71).

There are other molecules with changing levels of evidence (pre-clinical and clinical) for which promising results have been reported regarding their role as drug resistance biomarkers in OC and which represent a great utility in combinatorial therapy with specific inhibitors towards them in cases of platinum and PARPi drug resistance, with high/abnormal expression of these molecules: Mesothelin, Folate receptor alpha (FRα), Bloom syndrome RecQ helicase (BLM), cyclin E1 (CCNE1), miRNAs (MiR-181a), LncRNAs to cite the main examples (72–75).

In this context, cancer stem cell (CSC) signaling, epithelial-mesenchymal transition, miRNA, lncRNAs, and proteins involved in multiple biological processes are among the molecules that have shown promising results both *in vivo* and *in vitro*, and could be used as biomarkers of chemoresistance in OC (18–22, 76, 77), as shown by our results. Although, it is necessary to verify these findings in large groups of patients to determine the specificity, sensitivity, and feasibility of associating these biomolecules with relapse and response to therapy in patients.

Our most relevant results include a list of biomolecules that, according to our interpretation, has high potential as biomarkers of this phenomenon in OC. In that sense, we propose miRNAs, lncRNAs, and proteins (the most common ones) in this group. The first is BCL-2, which is a founding member of the BCL-2 protein family, has antiapoptotic activity and is overexpressed in various cancers. Several studies suggest that BCL-2 plays a principal role in cisplatin and paclitaxel resistance in OC (78–80). However, the underlying mechanism remains unknown. Xu et al. explained that BCL-2 overexpression confers cisplatin resistance and halves the rate of cisplatin-induced apoptosis in SKOV3 cells. In human SKOV3 OC xenografts, *BCL-2* attenuates the antitumor activity of cisplatin. Here, gene overexpression decreases tumor weight and volume three times after drug treatment. (78). Another study demonstrated that BCL-2 was upregulated in OC ascites cells and chemoresistant multicellular spheroids (MCSs). Interestingly, gene silencing or inhibition with siRNA or an ABT737 inhibitor enhanced cisplatin-induced apoptosis and reduced the inhibitory concentrations of cisplatin for MCS by 50% (81). Ying et al. found similar results for paclitaxel both *in vitro* and *in vivo.* The specific inhibition of BCL-2 overcomes paclitaxel resistance in both cases (79).

CHRF is a LncRNA that has been shown to be over-regulated in some cisplatin-resistant cellular models of OC (ES2, OVCAR3, and SKOV3) (with a relative expression 3 times higher). It is interesting that this finding was corroborated in patient samples with resistant disease and in an *in vivo* model. In addition, *CHRF* downregulation reverses cisplatin resistance (22). In contrast, Tan et al. reported a positive correlation between one lncRNA, CHRF, and one miRNA, miR-10b (22). In this regard, lncRNAs have acquired considerable importance as therapeutic targets and regulators of drug sensitivity in OC, and in the case of CHRF, several authors have highlighted their usefulness (82, 83).

SNAIL is a transcription factor that controls epithelial-mesenchymal transition, activating pluripotency-related genes, and its expression is associated with stem cell characteristics (and poor prognosis) in several cancers, such as breast, liver, ovarian, colorectal, and squamous cell carcinoma of the head and neck (84). This protein has been found to be over-regulated in recurrent glioblastoma (GBM) cancer tumors, promotes GBM cell resistance to Temozolomide-induced apoptosis. Therefore it is proposed as a biomarker of chemotherapy (85). Moreover, more mesenchymal OC lines are resistant to cisplatin (84). Kajiyama et al. obtained similar results, they found elevated SNAIL expression in a paclitaxel-resistant cell line compared with the control (86). Other studies have shown that SNAIL is involved in OC chemoresistance, supporting its utility as a biomarker (87, 88).

ALDH1is a CSC biomarker that modulates cell proliferation, stem cell differentiation, and resistance to chemotherapeutic agents (89). There is scientific evidence that prove both healthy and cancer cells with high levels of ALDH1 can serve as stem cells and have the capacity for self-renewal and stress resistance (27). Our results showed that in OC, a 3D model of serially passaged spheroids from tissue samples increased cisplatin resistance (the percentage of viable cells in 50 uM cisplatin was doubled in the last passage), *ALDH1A1* expression (fold changes around 10 in the last passage) and ALDH+ CSCs population. Moreover, treatment with an ALDH inhibitor (Compound 673A) substantially reduced the viability of cisplatin-resistant spheroids. *ALDH1* was on the top two over-regulated genes and was associated with chemoresistance (90). 974, a novel ALDH1A1 inhibitor, decreased the population of ovarian CSCs while suppressing platinum-induced senescence and stemness, accompanied by the downregulation of key stemness and chemoresistance pathways (91). Other studies have discovered that this protein may be useful in the diagnosis and treatment of chemoresistance in OC (92, 93).

Another molecule with high potential as a biomarker for chemoresistance is EZH2. It binds to the catalytic unit of polycomb repressive complex 2 (PRC2), which functions as a highly conserved histone methyltransferase that targets H3K27 to induce target gene silencing (94). According to our analysis, EZH2 plays a critical role in maintaining ovarian CSC stemness and chemoresistance, and patients with high EZH2 levels are more resistant to platinum drug treatment and have a worse prognosis (77). In this study by Wen et al., gradual increases (by western blot) in EZH2 levels were observed between cells not treated with cisplatin and cells with higher passages in the presence of the drug. In addition, the authors revealed a significant reduction in IC50 values for cisplatin-resistant CO cells after deletion of the gene coding for this protein, from an IC50 value of 7ug/ml to - 3 ug/ml. Other studies have demonstrated the predictive value of EZH2 in response to therapy in OC (94, 95). Because of its application in cancer progression and resistance to therapy, this protein is considered a new therapeutic target, with several inhibitors being developed and evaluated in clinical trials (96–98).

We believe that other molecules may be useful in the prediction of drug resistance in OC (miR-363, iASPP, and FZD7) also provide strong evidence in multiple study models (cell lines, tissue samples, xenografts) and make further analysis worthwhile (18, 20, 99–101). In relation to the latter, for example, it was one of the main over-regulated genes in several platinum-resistant cell models and xenografts. With fold-change greater than 8 between platinum-resistant SKOV3, OVCAR5 and OVCAR3 cells, as well as in platinum-treated SKOV3 and OVCAR3 xenografts. Interestingly, the population of FDZ7+ cells was enriched and increased in various platinum-resistant cell lines compared to the parental cells, which the authors suggest as a biomarker of persistence (20).

Despite the fact, we propose very interesting evidence of biomolecules, future investigation and validation trials involving a larger number of clinical samples and patients with sensitive and treatment-resistant diseases are needed. In general, we identified a lack of reproducibility of results by independent groups of scientists as well as a lack of continuity of results to determine the real usefulness of a molecule in the prediction of drug resistance, as noted by other authors who have analyzed the same situation (26). This is evidence of the great challenge scientists face in identifying resistance biomarkers in OC because aspects such as the selection process of a reproductive specimen, access to samples, heterogenicity, and histotypes must be considered, complicating obtaining results with a more universal application (102).

Our study contributes to the identification of biomarkers of drug resistance in OC, as it helps to the global analysis of related scientific literature via a bibliometric approach. It makes it possible to identify hotspots, trends, institutions, working groups, and other data and meta data that guide the related scientific community in planning new research and strategic collaboration networks. In such a way that soon, one or several molecules can be defined and will allow the monitoring of chemoresistance in OC in clinical practice in a concrete manner. We agree with other authors that it is necessary to take advantage of the results obtained thus far and propose research aimed at validating these molecules in tissue, tumor, and fluid samples and evaluating their predictive value in patient groups (103).

This study has several limitations that are related to the use of bibliometric analysis instruments. Another one limitation is related to the source of the documentation used since we considered only documents from the PubMed database for a restricted period. We did not cover all the scientific literature related to drug resistance biomarkers in OC.

## 5 Conclusions and perspectives

Currently, OC is highly lethal for two main reasons: late diagnosis and relapse because of drug resistance. Therefore, scientific research in both areas is a global priority. Although this study showed that the scientific community is working on chemoresistance, it is important to notice that it is a modern and emerging area of interdisciplinary relevance. Research in the area is led by the United States and China and covers 4 thematic groups involving basic science studies, prognostic approaches, drug resistance, and more recently, the search for resistance biomarkers. While there are encouraging findings regarding possible resistance biomarkers, a more thorough examination of the current state of the art is needed to guide future research toward identifying and validating resistance biomarkers with high predictive value in OC. In addition, it is necessary to conduct future studies to determine the effectiveness of various molecules in monitoring and predicting chemoresistance. Efforts must aim at biomarker testing in larger cohorts in randomized trials and studies that also consider the histopathological divergence of the disease.

## Data Availability

The original contributions presented in the study are included in the article/supplementary material, further inquiries can be directed to the corresponding author/s.
